# Genus *Sambucus*: Exploring Its Potential as a Functional Food Ingredient with Neuroprotective Properties Mediated by Antioxidant and Anti-Inflammatory Mechanisms

**DOI:** 10.3390/ijms25147843

**Published:** 2024-07-18

**Authors:** Anna Merecz-Sadowska, Przemysław Sitarek, Karolina Zajdel, Wiktoria Sztandera, Radosław Zajdel

**Affiliations:** 1 Department of Economic and Medical Informatics, University of Lodz, 90-214 Lodz, Poland; anna.merecz-sadowska@uni.lodz.pl; 2 Department of Allergology and Respiratory Rehabilitation, Medical University of Lodz, 90-725 Lodz, Poland; 3 Department of Medical Biology, Medical University of Lodz, Muszynskiego 1, 90-151 Lodz, Poland; przemyslaw.sitarek@umed.lodz.pl; 4 Department of Medical Informatics and Statistics, Medical University of Lodz, 90-645 Lodz, Poland; karolina.smigiel@umed.lodz.pl; 5 Department of Internal Medicine, Rehabilitation and Physical Medicine, Medical University of Lodz, 90-647 Lodz, Poland; wiktoria.sztandera@umed.lodz.pl

**Keywords:** *Sambucus nigra*, elderberry, neuroprotection, cognitive function, oxidative stress, neuroinflammation, functional food

## Abstract

The genus *Sambucus*, mainly *Sambucus nigra*, has emerged as a valuable source of bioactive compounds with potential neuroprotective properties. This review explores the antioxidant, anti-inflammatory, and neuroregenerative effects of *Sambucus*-derived compounds and their implications for brain health and cognitive function. In vitro studies have demonstrated the ability of *Sambucus* extracts to mitigate oxidative stress, modulate inflammatory responses, and promote neural stem cell proliferation and differentiation. In vivo studies using animal models of neurodegenerative diseases, such as Alzheimer’s and Parkinson’s, have shown that *Sambucus* compounds can improve cognitive function, motor performance, and neuronal survival while attenuating neuroinflammation and oxidative damage. The neuroprotective effects of *Sambucus* are primarily attributed to its rich content of polyphenols, particularly anthocyanins, which exert their benefits through multiple mechanisms, including the modulation of signaling pathways involved in inflammation, apoptosis, mitochondrial function, and oxidative stress. Furthermore, the potential of *Sambucus* as a functional food ingredient is discussed, highlighting its application in various food products and the challenges associated with the stability and bioavailability of its bioactive compounds. This review provides a comprehensive overview of the current state of research on the neuroprotective potential of *Sambucus* and its derivatives, offering valuable insights for the development of dietary strategies to promote brain health and prevent age-related cognitive decline.

## 1. Introduction

The genus *Sambucus* L., belonging to the family *Viburnaceae*, comprises approximately 29 recognized species, predominantly deciduous shrubs, perennial herbs, or small trees. The dispersal of *Sambucus* has followed two distinct routes, covering an area extending west to Europe, North America, South America, and northern Asia, and east to Southeast Asia and Australia [1,2]. *Sambucus* species exhibit distinctive morphological characteristics: the leaves are typically compound, pinnate to ovate–lanceolate, or ovate–elliptic with serrated margins, and the inflorescences are terminal, flat-topped umbellate or corymbose cymes, which can be pyramidal paniculate. The fruits are small, rounded berry-like drupes, ranging from 4 to 7 mm in diameter, with varying colors at maturity. Each fruit contains three to five pyrenes and three to five triquetrous or ellipsoid seeds [3,4]. Despite these common features, the taxonomy of *Sambucus* remains challenging due to the morphological and genetic variations within and between species, as well as the variability among their widespread congeners [5].

*Sambucus* species have been found to possess significant pharmacological properties [6,7] which allow for the treatment of a wide range of ailments such as musculoskeletal disorders, metabolic diseases, respiratory diseases and skin conditions [8,9,10,11]. Moreover, the plants are rich sources of bioactive compounds, with about 425 secondary metabolites identified, most of which are phenolic compounds and terpenoids [2]. These constituents have been associated with significant antioxidant and anti-inflammatory activities, among others [12,13,14,15]. 

**Figure 1:**
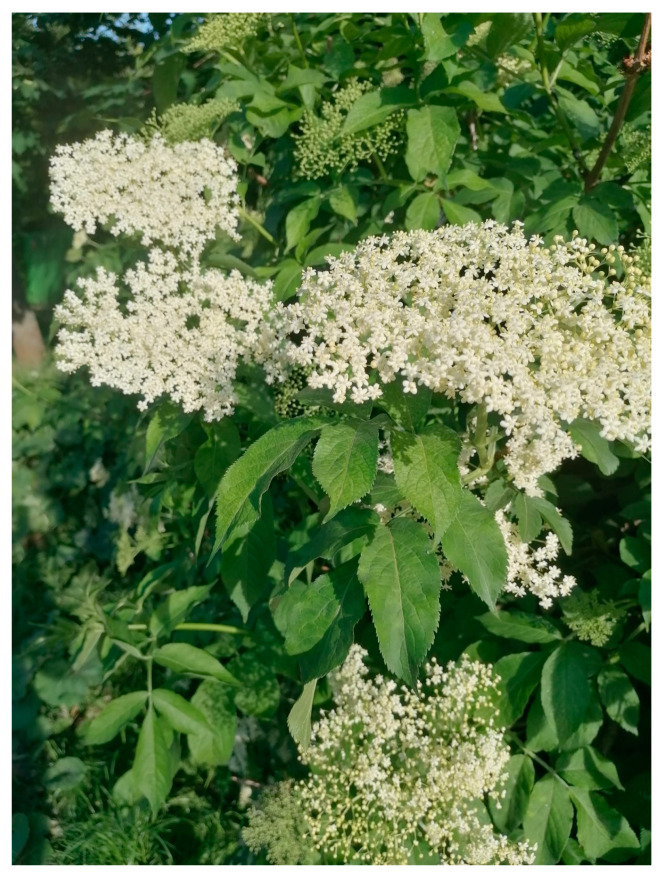
The image of *S. nigra*.

One of the most extensively studied species is *Sambucus nigra* L. (black elder) (Figure 1). It has a long history of use as a food source. Among the various parts of the plant, the flowers and berries are of particular interest due to their rich bioactive compound content and their potential health benefits. Elderflowers contain a range of volatile compounds, mainly of terpenoid origin, such as monoterpenes (α-phellandrene, α-terpinene, limonene, 1,8-cineole, β-ocimene, γ-terpinene, β-ocimene, terpinolene), terpenoid alcohols and oxides (linalool oxide, cis-rose oxide, trans-rose oxide, cis-linalool oxide, nerol oxide, linalool, hotrienol, α-terpineol, citronellol, nerol, geraniol), and a sesquiterpene (β-caryophyllene) [16,17]. According to a study by Ağalar et al., the total terpenoid content in the elderflower n-hexane extract was 84.4%. In the essential oil, terpenoids constituted 90.4% of the total volatile compounds identified [18]. Elderflowers are also rich in phenolic compounds, particularly flavonol glycosides like quercetin-3-*O*-rutinoside, kaempferol-3-*O*-rutinoside, and isorhamnetin-3-*O*-rutinoside, which constitute over 90% of the total flavonoid content in most elderflower genotypes. Additionally, 5-*O*-caffeoylquinic acid (chlorogenic acid) and 1,5-di-*O*-caffeoylquinic acid are the major phenolic acids present in elderflowers, comprising over 70% of the total phenolic acid content. Elderberries, on the other hand, are known for their high anthocyanin content. The main anthocyanins found in elderberries are cyanidin-3-sambubioside and cyanidin-3-glucoside, with lower amounts of cyanidin-3-sambubioside-5-glucoside, cyanidin-3,5-diglucoside, and cyanidin-3-rutinoside [16,17]. According to the study by Nurzyńska-Wierdak et al. the total anthocyanin content in elderberry fruit was found to be 0.29 g per 100 g of dry matter [19]. Elderberries also contain other phenolic compounds such as chlorogenic acid, neochlorogenic acid, cryptochlorogenic acid, quercetin-3-rutinoside, quercetin-3-glucoside, kaempferol-3-rutinoside, isorhamnetin-3-rutinoside, and isorhamnetin-3-glucoside. Based on a review by Pascariu and Israel-Roming, the total phenolic content in elderberry fruits varied widely across studies, ranging from 516 to 8974 mg/100 g dry weight [20]. In terms of lipophilic compounds, elderberries are rich in triterpenoids, particularly ursolic and oleanolic acids [21].

The central nervous system (CNS) is subject to various neuronal injuries caused by acute or chronic neurodegenerative diseases. These disorders result from the breakdown and deterioration of neurons in the CNS, often leading to impaired cognitive and intellectual faculties [22,23]. Neurodegenerative diseases are influenced by a range of risk factors, particularly aging and various genetic and environmental factors. As people age, they are more subject to oxidative stress, inflammation, and loss of neurotransmitters; these are all common pathological features of neurodegenerative diseases [24].

Extracts derived from the *Sambucus* genus are believed to exhibit remarkable anti-aging and neuroprotective properties. These have been attributed to the presence of high levels of polyphenolic constituents in their extracts, which are believed to exert neuroprotective effects by various mechanisms including antioxidant and anti-inflammatory activities. In addition, their potent neuroprotective capabilities against complex neurodegenerative processes may be realized by the multi-target effects exhibited by these compounds [25].

The aim of this paper is to review the current state of research regarding the neuroprotective potential of bioactive compounds derived from the *Sambucus* genus, mainly *Sambucus nigra*, in the context of their dietary applications and effects on human neurocognitive function. It explores the antioxidant, anti-inflammatory, anti-aging, and neuroregenerative properties of *Sambucus*-derived compounds, and to investigate their potential applications as functional food ingredients to promote brain health and cognitive performance. By examining the neuroprotective effects of *Sambucus* extracts and their bioactive compounds in various models of neurodegenerative diseases, this review seeks to contribute to the growing body of evidence supporting the use of dietary interventions as a promising strategy for preventing age-related cognitive decline and promoting optimal neurocognitive function.

## 2. Study Design

A systematic literature search was conducted using Web of Science, PubMed, and Scopus databases. The search strategy employed the following keywords and their combinations: ‘*Sambucus*’, ‘elderberry’, ‘elderflower’, ‘antioxidant’, ‘anti-inflammatory’, ‘neuroprotection’, and ‘neurodegenerative diseases’, The search was limited to peer-reviewed articles published in English between 2004 and 2024.

The literature search yielded a substantial body of research on the neuroprotective properties of *Sambucus*-derived compounds. Studies encompassed a wide range of topics, including the identification and characterization of bioactive compounds, their antioxidant and anti-inflammatory effects, neuroprotective mechanisms, and potential applications in neurodegenerative diseases. The collected data provided insights into both in vitro and in vivo studies, as well as the potential use of *Sambucus* extracts in functional foods. To quantify the research trends in this field and to understand the evolving scientific interest, a comprehensive bibliometric analysis was conducted.

**Figure 2:**
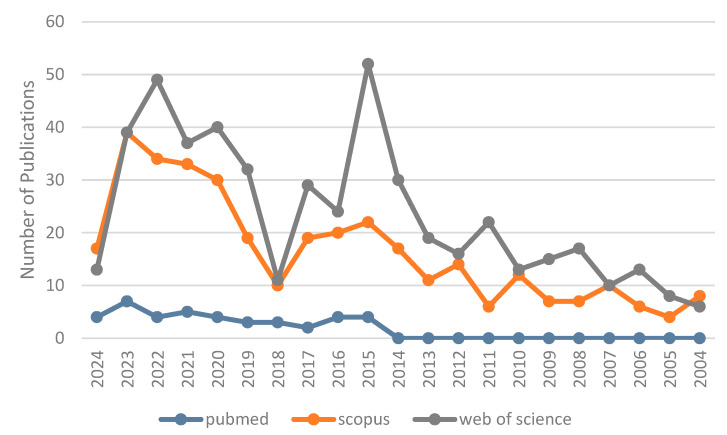
Temporal trend of scientific publications on neuroprotective properties of *Sambucus*-derived compounds (2004–2024). The graph illustrates the annual number of publications indexed in the Web of Science database over a 20-year period, demonstrating a marked increase in research interest, particularly in recent years.

A comprehensive bibliometric analysis was conducted using three major scientific databases: PubMed, Scopus, and Web of Science. The analysis covered publications from 2004 to 2024, providing a two-decade perspective on research trends related to the neuroprotective properties of *Sambucus*-derived compounds (Figure 2).

The Web of Science database demonstrated the highest overall publication numbers. The annual publication count ranged from 6 (in 2004) to 52 (in 2015). Notably, there was a significant surge in publications from 2015 onwards, with annual counts frequently exceeding 30. The years from 2020–2023 were particularly productive, with annual publications consistently above 37, peaking at 49 in 2022.

Across all three databases, a clear trend of increasing research interest is evident, particularly in the last five years. This trend is most pronounced in Scopus and Web of Science databases, which show a substantial rise in publications from 2018 onwards. The data from 2024, although partial, suggests a continuation of this upward trend.

This bibliometric analysis underscores the growing scientific interest in the neuroprotective properties of *Sambucus*-derived compounds, reflecting the increasing recognition of their potential in addressing neurodegenerative disorders.

## 3. Oxidative Stress, Free Radicals, and Antioxidant Compounds Derived from *Sambucus* Plants: Implications for Neuroprotection

Oxidative stress is a condition characterized by an imbalance between the production of reactive oxygen species (ROS) and the ability of the body’s antioxidant defense systems to counteract their harmful effects. ROS are highly reactive molecules that are generated as byproducts of normal cellular metabolism, particularly during the process of oxidative phosphorylation in the mitochondria [26]. These species include free radicals such as superoxide anions (O_2_•^−^), hydroxyl radicals (•OH), and non-radical molecules like hydrogen peroxide (H_2_O_2_). Under normal physiological conditions, ROS play an essential role in cell signaling and homeostasis. However, when ROS levels become excessively high, they can cause damage to cellular components such as lipids, proteins, and DNA, leading to oxidative stress [27]. This oxidative damage has been implicated in the pathogenesis of various neurodegenerative diseases, including Alzheimer’s disease (AD) and Parkinson’s disease (PD) [28].

The *Sambucus* genus, mainly *S. nigra*, has been recognized as a valuable source of antioxidant compounds that can help combat oxidative stress. The main bioactive components found in *S. nigra* are polyphenols [29,30]. These polyphenolic compounds possess potent antioxidant properties due to their ability to scavenge free radicals, donate hydrogen atoms, and chelate metal ions [31]. Numerous studies have demonstrated the antioxidant capacity of *S. nigra* extracts in vitro, based on oxygen radical absorbance capacity (ORAC) and the 2,2-diphenyl-1-picrylhydrazyl (DPPH) radical scavenging methods [13,32,33,34]. Moreover, in vitro studies using neuronal cell lines have shown that *S. nigra* extracts can effectively protect cells against oxidative stress-induced damage by reducing intracellular ROS levels, preventing glutathione depletion, and modulating the activity of antioxidant enzymes like glutathione peroxidase and glutathione reductase [34,35]. Natural compounds, including extracts derived from *S. nigra*, possess antioxidant properties and may have neuroprotective potential by mitigating oxidative stress in the brain [36].

Compared to other organs, the brain is particularly vulnerable to reactive oxygen species due to its high oxygen consumption, abundant lipid content, and relatively weak antioxidant defenses, as well as limited regenerative capacity [37]. Oxidative stress may trigger molecular pathways leading to the progressive loss of neuronal structures and functions and is considered a major factor in the development of neurodegenerative disorders [38,39]. In the case of AD, several key observations indicate that oxidative stress may play a key role in its etiology. For example, the levels of oxidized nucleic acids, proteins, and lipids tend to be elevated in AD brains, and such changes also appear earlier than other pathological manifestations; in addition, high amounts of metal ions are present, which can catalyze free radical generation, accompanied by high levels of advanced glycation end products (AGEs). Also, the condition is characterized by the presence of amyloid-β (Aβ) peptides, which can directly and indirectly induce cellular oxidative stress. Aβ aggregates can interact with cell membranes and lead to intracellular ROS accumulation, causing lipid peroxidation, membrane disintegration, and ultimately cell lysis. Furthermore, Aβ can indirectly generate an oxidative microenvironment through the induction of a local immune response and inflammation [40].

Similarly, free radicals have been implicated in the pathogenesis of PD. PD patients are characterized by elevated oxidative stress, which is thought to be a result of multiple factors, including mitochondrial dysfunction, iron dysregulation, inflammation, and impaired antioxidant defense systems. While the loss of dopaminergic neurons is a key feature of PD, it is not the sole contributor to increased oxidative stress in the disease [41]. Increased levels of ROS are also associated with dopamine metabolism, and dopamine-associated oxidative stress may contribute to the inflammatory reaction seen in PD [42].

Several studies have investigated the antioxidant capacity and neuroprotective potential of bioactive compounds derived from *Sambucus* plants, particularly in the context of oxidative stress. Neves et al. [43], May et al. [44] and Palomino et al. [34] focused on the antioxidant effects of elderberry extracts and their bioactive compounds on human SH-SY5Y neuroblastoma cells, which are widely used as a model to study neurodegenerative diseases and oxidative stress-induced neuronal damage. Neves et al. [43] explored the antioxidant capacity and neuroprotective potential of an anthocyanin-enriched extract. The findings demonstrate that the extract has significant antioxidant activity and affinity for mitochondrial membranes. Elderberry extract protected against rotenone-induced cytotoxicity, modulated cell redox state, and reduced the increase in intracellular ROS levels induced by rotenone. Furthermore, the extract increased the activity of antioxidant enzymes, such as superoxide dismutase, glutathione peroxidase, and glutathione reductase, as well as the activity of mitochondrial respiratory complexes I and II. The authors suggest that elderberry extract may have neuroprotective effects resulting from the intrinsic antioxidant and mitochondria-modulating properties of its anthocyanins. Similarly, May et al. [44] investigated the antioxidant capacity, phenolic content, and neuroprotective potential of elderberry extract. The study examined the total phenolic, flavonoid, and anthocyanin content of the extract, as well as its oxygen radical absorbance capacity (ORAC), nitrogen radical scavenging capacity (DPPH) and metal-chelating capacities (Cu^2+^ and Fe^2+^). The results show that pre-treatment with an elderberry extract has neuroprotective effects, reflected in it significantly preventing the reduction in cell viability induced by H_2_O_2_. Furthermore, elderberry extract increases cell viability when administered as a treatment after exposure to H_2_O_2_, suggesting potential therapeutic effects. In another study, Palomino et al. [33] evaluated the antioxidant capacity and neuroprotective potential of various extracts. The aqueous and ethanolic extracts exhibited higher antioxidant activity compared to the methanolic extract. Both aqueous and ethanolic extracts significantly reduced basal ROS levels and attenuated tert-butylhydroperoxide (t-BOOH)-induced ROS overproduction in a dose-dependent manner. The extracts also partially protected against t-BOOH-induced glutathione depletion and modulated the activities of glutathione peroxidase (GPx) and glutathione reductase (GR).

Simonyi et al. [45] and Jiang et al. [46] both investigated the antioxidant effects of elderberry extracts and their bioactive compounds on murine BV-2 microglial cells; these play a crucial role in the immune response of the central nervous system and are involved in various neurodegenerative disorders. More precisely, Simonyi et al. [45] investigated the antioxidant effects of extracts prepared from elderberry pomace, as well as some of the anthocyanins (cyanidin chloride and cyanidin 3-*O*-glucoside) and flavonols (quercetin and rutin) present in elderberry. The ethanolic elderberry extracts effectively inhibited ROS production induced by lipopolysaccharide (LPS) or interferon gamma (IFNγ) in a dose-dependent manner. The anthocyanins cyanidin chloride and cyanidin 3-O-glucoside strongly inhibited ROS production in microglial cells. Among the flavonols tested, quercetin was found to be the most potent component in mitigating oxidative stress in microglial cells. Similarly, Jiang et al. [46] examined the effects of elderberry juice extracts of different genotypes on oxidative responses following LPS or IFNγ stimulation. The ‘Wyldewood’ extract exhibited antioxidant properties by inhibiting IFNγ-induced ROS production and suppressing the expression of phosphorylated extracellular signal-regulated kinase 1/2 (p-ERK1/2), a key mediator of ROS generation in microglial cells. The authors highlight the complex and genotype-specific effects of elderberry extracts on oxidative pathways in microglial cells.

Additionally, Aurélie de Rus Jacquet et al. [47] investigated the antioxidant properties of Sambucus caerulea flower extract in primary rat cortical astrocytes. They demonstrated that elderflower extract significantly activated the Nrf2/ARE antioxidant pathway in cortical astrocytes. A similar effect was observed in astrocytes derived from human induced pluripotent stem cells. Moreover, a trend towards increased Nrf2-mediated transcription was observed in astrocytes within mixed neuron-glia cultures from rat midbrain following elderflower extract treatment. These results suggest that elderflower extract can effectively activate the antioxidant response in cortical astrocytes, which play a crucial role in supporting and protecting neurons in the central nervous system. The ability of elderflower extract to stimulate the Nrf2 pathway in various types of astrocytes underscores its potential as a neuroprotective agent, possibly by enhancing cellular defense mechanisms against oxidative stress in the brain.

**Figure 3:**
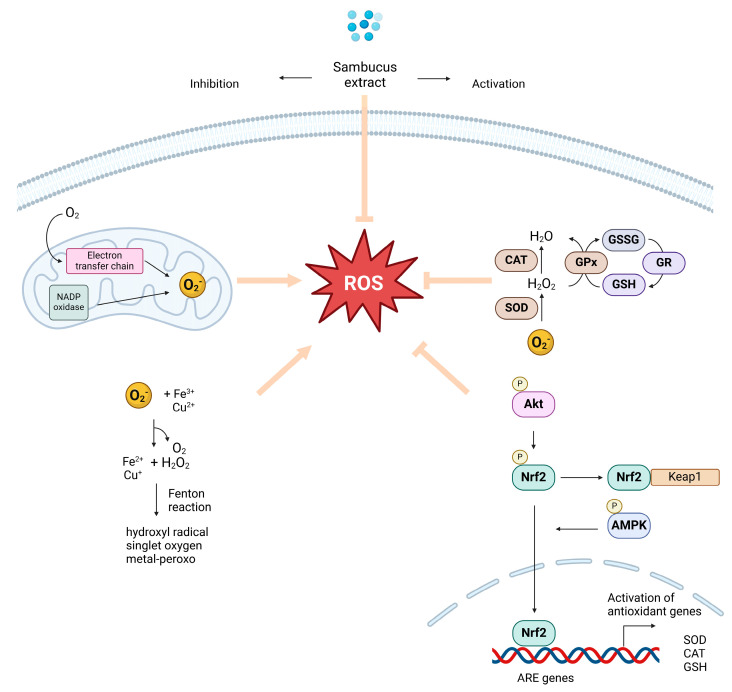
Schematic representation of potential antioxidant action of *Sambucus*-derived compounds.

The *Sambucus* genus, mainly *S. nigra*, has emerged as a promising natural source of potent antioxidant compounds that can effectively mitigate oxidative stress in the brain (Figure 3). Numerous in vitro studies on cell lines have found these extracts to effectively protect cells against oxidative stress-induced damage. Moreover, the patent WO 2007/113892 A2 discloses a method for obtaining an antioxidant-rich extract from the fruits of *S. nigra*. The patented process involves drying the fruits, extracting them with an aqueous alcoholic solvent, and purifying the extract to increase its anthocyanin content. The resulting extract rich in anthocyanin highlights the potential of *S. nigra* as a natural source of antioxidants with potential applications in various fields where oxidative stress is a concern.

## 4. Inflammation, Inflammatory Mediators, and Anti-Inflammatory Compounds Derived from *Sambucus* Plants: Implications for Neuroprotection

Inflammation is a complex biological response to harmful stimuli, such as pathogens, damaged cells, or irritants with the aim of removing the injurious stimuli and initiating the healing process. The inflammatory process involves a cascade of biochemical events that propagate and mature the inflammatory response; these involve the local vascular system, the immune system, and various cells within the injured tissue. The cardinal signs of inflammation are heat, pain, redness, swelling, and loss of function. Inflammation can be acute, i.e., the initial response to harmful stimuli, achieved by increased movement of plasma and leukocytes from the blood into the injured tissues; it can also be chronic, inducing a progressive change in the type of cells present at the site of inflammation, and characterized by simultaneous destruction and healing of tissue [48,49].

The inflammatory response is tightly regulated by a variety of mediators produced by various cells, including macrophages, dendritic cells, mast cells, neutrophils, and lymphocytes. The inflammatory response can be triggered by a variety of stimuli, including pathogen-associated molecular patterns (PAMPs) and damage-associated molecular patterns (DAMPs). PAMPs are molecules associated with pathogens such as LPS, peptidoglycan, and double-stranded RNA. DAMPs are molecules released from damaged cells, such as ATP, DNA, and heat-shock proteins. Recognition of PAMPs and DAMPs by pattern recognition receptors (PRRs) in immune cells leads to the activation of signaling pathways that result in the production of inflammatory mediators [50,51].

Inflammatory mediators play a crucial role in the initiation, amplification, and resolution of the inflammatory response. The activation of macrophages and other immune cells results in the release of a variety of inflammatory mediators, including cytokines, chemokines, and lipid mediators. Interleukins, such as IL-1β, IL-6, and IL-8, are produced by activated macrophages and play a key role in the acute phase response, as well as neutrophil recruitment to the site of inflammation. Tumor necrosis factor alpha (TNF-α) is another important pro-inflammatory cytokine that is produced by activated macrophages [52,53].

Prostaglandin E2 (PGE2) is a lipid mediator produced by the enzyme cyclooxygenase-2 (COX-2) and plays a key role in the induction of fever, pain, and vasodilation [54]. Another group of lipid mediators, leukotrienes, are also involved in the inflammatory response. Leukotrienes are produced by the 5-lipoxygenase pathway and are potent chemo-attractants for neutrophils, eosinophils, and monocytes. They also contribute to vascular permeability, bronchoconstriction, and mucus secretion [55]. Reactive nitric oxide (NO) is also an important inflammatory mediator that is produced by activated macrophages and neutrophils. NO is synthesized by nitric oxide synthases (NOS), particularly the inducible isoform (iNOS), which is rapidly induced following an inflammatory stimulus. Although NO plays a crucial role in the defense mechanism, elevated concentrations or persistent inflammation can cause cellular damage and chronic inflammatory disease [56]. ROS are also important inflammatory mediators [57]. Natural compounds, such as polyphenols, have been shown to possess anti-inflammatory activity by modulating the production of these inflammatory mediators; this modulation is essential for resolving inflammation and preventing chronic inflammatory diseases. Among these, ROS play a dual role in inflammatory processes, serving both as signaling molecules and exerting oxidative stress when present at high levels [58,59].

Chronic inflammation has been increasingly recognized as a key factor in the pathogenesis of neurodegenerative diseases, such as AD and PD. In these disorders, the persistent activation of resident immune cells in the CNS, particularly microglia and astrocytes, as well as the infiltration of peripheral immune cells, contribute to neuronal damage and exacerbate disease progression. Microglia, the resident macrophages of the CNS, are the first line of defense against pathogens, damaged cells, and protein aggregates [60]. In the healthy brain, microglia exist in a resting state, continuously surveying their microenvironment for signs of danger. Upon detection of harmful stimuli, such as Aβ or α-synuclein, microglia become activated and undergo morphological and functional changes. In chronic neurodegenerative diseases, microglia can become persistently activated, leading to a self-perpetuating cycle of inflammation and neuronal loss [61,62]. In addition, when exposed to inflammatory stimuli, astrocytes undergo a process called reactive astrogliosis, characterized by hypertrophy, proliferation, and increased expression of glial fibrillary acidic protein (GFAP). Reactive astrocytes produce pro-inflammatory cytokines, chemokines, NOS and ROS, thus contributing to the inflammatory milieu in the CNS. Additionally, astrocytes can modulate the activity of microglia through the release of factors such as ATP, glutamate, and cytokines, further amplifying the inflammatory response. The crosstalk between microglia and astrocytes is essential in the regulation of neuroinflammation. For example, activated microglia can induce reactive astrogliosis through the release of cytokines such as IL-1β and TNF-α, while astrocytes can, in turn, modulate microglial activity through the production of chemokines and neurotrophic factors [63,64].

In AD, the accumulation of Aβ peptide and neurofibrillary tangles composed of hyperphosphorylated tau protein triggers an inflammatory response in the brain. Activated microglia and astrocytes surrounding senile plaques produce pro-inflammatory cytokines, such as IL-1β, TNF-α, and IL-6, as well as chemokines and prostaglandins, which can promote neuronal death [65]. Similarly, in PD, the aggregation of α-synuclein and the loss of dopaminergic neurons in the substantia nigra are associated with microglial activation and increased production of pro-inflammatory mediators, which contribute to the progression of the disease [66].

Several in vitro studies have investigated the anti-inflammatory properties of *S. nigra* extracts and their bioactive components, particularly in the context of macrophage and neutrophil function. Ho et al. [67], Santin [68], and Olejnik et al. [69] examined the effects of elderberry extracts on LPS-stimulated RAW 264.7 macrophages and neutrophils, a widely used cell line for studying inflammatory responses. Ho et al. [67] investigated the effects of *S. nigra* fruit and flower extracts, as well as their various constituent polyphenols, on LPS-stimulated RAW 264.7 macrophages. The results showed that the juice inhibited the secretion of pro-inflammatory factors by macrophages by 30%, and the acidified methanol extract by 50%. Among the polyphenolic compounds, cyanidins, cyanidin-3-glucoside, and cyanidin-3-glucoside sambubioside reduced the secretion of pro-inflammatory factors by about 60% to 70%, while quercetin decreased the secretion by 80% and chlorogenic acid by 51.5%. Santin et al. [68] investigated the anti-inflammatory properties of the *S. nigra* flower extract. The extract reduced the levels of pro-inflammatory cytokines, such as TNF-α, IL-1β, and IL-6, in neutrophils and COX-2 macrophages stimulated by LPS while increasing the production of anti-inflammatory IL-10. The authors suggest that rutin, a major component of the extract, plays a crucial role in the observed anti-inflammatory effects. Similarly, Olejnik et al. [69] reported that elderberry extracts subjected to simulated gastrointestinal digestion produced anti-inflammatory effects in LPS-stimulated RAW 264.7 cells by downregulating the pathways that produce IL-1β, IL-6, TNF-α, COX-2, PGE2, and NO. These findings suggest that elderberry extracts and their bioactive components can effectively modulate inflammatory responses in macrophages and neutrophils by reducing the production of key pro-inflammatory mediators.

In addition to macrophages, the anti-inflammatory effects of elderberry extracts have also been investigated in microglial cells, which are the resident immune cells of the central nervous system. Jiang et al. [46] and Simonyi et al. [45] examined the effects of elderberry on murine BV-2 microglial cells, a widely used cell line for studying neuroinflammation in the brain. Jiang et al. [46] evaluated the effect of elderberry juice extracts from different genotypes on inflammatory responses in BV-2 cells stimulated with LPS or IFNγ. Most elderberry juice extracts exerted minimal or no inhibitory effects on LPS-induced NO production, and surprisingly, some extracts, particularly from the ‘Wyldewood’, ‘Ozone’, and ‘Sperandio’ genotypes, caused a significant increase in NO production upon stimulation with IFNγ. The ‘Wyldewood’ extract also enhanced IFNγ-induced iNOS protein expression. These findings highlight the complex and genotype-specific effects of elderberry extracts on oxidative and inflammatory pathways in microglial cells. Similarly, Simonyi et al. [45] investigated the antioxidant effects of extracts prepared from elderberry pomace, as well as some of the anthocyanins (cyanidin chloride and cyanidin 3-*O*-glucoside) and flavonols (quercetin and rutin) present in elderberry, on BV-2 microglial cells. The elderberry ethanol extracts effectively inhibited LPS or IFNγ-induced ROS production in a dose-dependent manner. The anthocyanins cyanidin chloride and cyanidin 3-*O*-glucoside strongly inhibited ROS production in microglial cells. Among the flavonols tested, quercetin emerged as the most potent component in mitigating oxidative stress in microglial cells.

Furthermore, *S. nigra* fruit extract has also exhibited promising anti-inflammatory properties in vivo. In the cotton pellet-induced granuloma test, oral administration of extract produced a dose-dependent anti-inflammatory response, with the highest dose significantly reducing the granuloma weight by 28.4%, which was comparable to the effect of the reference drug diclofenac; these anti-inflammatory effects were further supported by histopathological examination of the granulomatous tissue. These results suggest that the polyphenol-rich *S. nigra* fruit extract, particularly abundant in anthocyanins, may have potential as a natural anti-inflammatory agent, most likely through mechanisms involving the inhibition of inflammatory mediators and enzymes such as COX [70]. In addition, a study by Santin et al. [68] investigated the anti-inflammatory effects of a lyophilized aqueous extract obtained from *S. nigra* flowers in vivo. Oral treatment with the extract lowered carrageenan-induced mechanical hypersensitivity in male Swiss mice with inflammation induced by carrageenan, suggesting it may have analgesic effects.

In addition to their direct anti-inflammatory effects, recent research has also explored the potential of elderberry extracts to modulate the gut–brain axis and its implications for neuroinflammation and cognitive function. Namakin et al. [71] investigated the therapeutic effects of an elderberry diet on the dysfunction of the gut–brain axis, neuroinflammation, and cognitive impairment in a rat model of irritable bowel syndrome (IBS). IBS was induced using intracolonic instillation of acetic acid, and a diet enriched with elderberry extract was administered for eight consecutive weeks. The elderberry diet improved locomotion and decreased anxiety-like behavior; it also reduced the expression of the pro-inflammatory cytokine TNF-α in colon tissue, increased the thickness of the mucosal layer and increased the number of goblet cells in the colon, suggesting that it may have a protective effect on the intestinal barrier and mucosal integrity. The elderberry diet also prevented astrogliosis and astrocyte reactivity in the hippocampus of the IBS rats and protected both cortical and hippocampal neurons from degeneration. The authors attribute the beneficial effects of elderberry to its antioxidant and immunomodulatory properties, which may counteract gut-induced neuroinflammation and cognitive impairment associated with IBS.

**Figure 4:**
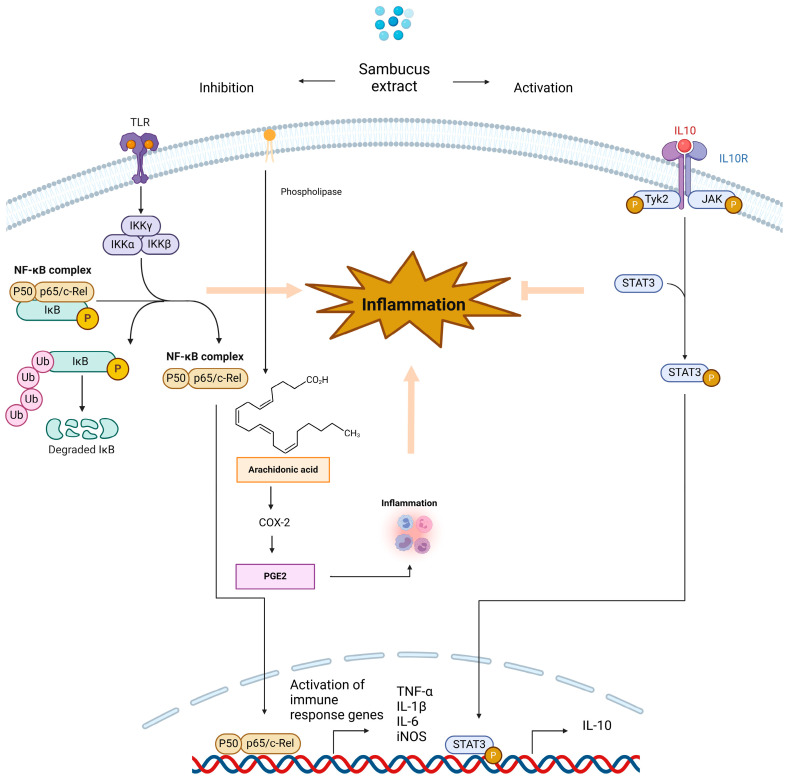
Schematic representation of potential anti-inflammatory action of *Sambucus*-derived compounds.

The *Sambucus* genus, mainly *S. nigra*, has emerged as a promising natural source of potent anti-inflammatory compounds that can effectively mitigate neuroinflammation in the brain (Figure 4). Numerous studies on cell lines have shown that these extracts can effectively modulate inflammatory responses by reducing the production of pro-inflammatory compounds. Furthermore, elderberry extracts have demonstrated the ability to attenuate the activation of microglial cells and astrocytes, which are key players in the neuroinflammatory process associated with neurodegenerative disorders. The preparation of elderberry fruit extract using a specific extraction process and its use in the treatment of inflammatory conditions has been recorded in patent number WO2011144639A1. Elderberry extract has been shown to inhibit pro-inflammatory cytokine production (IL-1β, IL-6, and TNF-α) and COX-2 and iNOS expression in LPS-stimulated macrophages. Furthermore, the extract demonstrated anti-inflammatory effects in an in vivo model of carrageenan-induced paw edema in rats, reducing paw swelling and inflammatory cell infiltration. These findings underscore the potential of *S. nigra* as a natural source of anti-inflammatory agents with promising applications in the prevention and treatment of inflammatory conditions.

## 5. Aging, Age-Related Neurodegeneration, and Anti-Aging Compounds Derived from *Sambucus* Plants

Aging is an inevitable and complex process characterized by a progressive decline in physiological function and an increased susceptibility to disease and death. While the biological mechanisms underlying the aging process are not fully understood, several hallmarks of aging have been identified, including genomic instability, telomere attrition, epigenetic alterations, loss of proteostasis, deregulated nutrient sensing, mitochondrial dysfunction, cellular senescence, stem cell exhaustion, and altered intercellular communication. These hallmarks are interconnected and contribute to the functional deterioration of tissues and organs over time [72,73]. The brain is particularly vulnerable to the effects of aging, as it is primarily composed of postmitotic cells, such as neurons and oligodendrocytes, which are more susceptible to accumulated damage. The age-related changes in the brain are thought to create a permissive environment for the development of neurodegenerative diseases, which are characterized by the progressive loss of specific neuronal populations and the accumulation of toxic protein aggregates [74].

The connection between aging and neurodegenerative diseases is complex and multifaceted. Aging is the primary risk factor for most neurodegenerative disorders, including AD and PD. The prevalence of these diseases increases dramatically with age and are rarely observed in younger individuals. This strong association suggests that the underlying mechanisms of aging may play a crucial role in the onset and progression of neurodegeneration. As the brain ages, it becomes increasingly vulnerable to the accumulation of toxic protein aggregates, such as amyloid-β plaques and neurofibrillary tangles in AD and α-synuclein-containing Lewy bodies in PD. Additionally, the aging brain exhibits a progressive decline in the efficiency of cellular maintenance and repair processes, leading to the accumulation of oxidative damage, mitochondrial dysfunction, and chronic inflammation. These age-related changes create a permissive environment for the development of neurodegenerative pathologies. Furthermore, the loss of proteostasis, impaired autophagy, and cellular senescence associated with aging may contribute to the selective vulnerability of specific neuronal populations to various neurodegenerative diseases [24].

Anthocyanins extracted from the fruits of *S. canadensis* have been shown to possess anti-aging properties both in vivo and in vitro. A study by Hu et al. [75] investigated the molecular mechanisms underlying the anti-aging effects of these anthocyanins, focusing on cellular senescence and the PI3K/AKT/mTOR signaling pathway. The study revealed that anthocyanins significantly reduced cell senescence and aging of the lens in mice by inhibiting the activity of the PI3K/AKT/mTOR signaling pathway. This inhibition led to the promotion of apoptosis in senescent cells, increased autophagic and mitophagic flux, and enhanced the renewal of mitochondria and cells, ultimately maintaining cellular homeostasis and attenuating aging. The study’s findings suggest that anthocyanins may act as senolytics, i.e., compounds that selectively eliminate senescent cells, which are known to accumulate with age and contribute to age-related diseases. By targeting the PI3K/AKT/mTOR pathway, which plays a central role in regulating cell proliferation, apoptosis, and autophagy, anthocyanins were able to modulate key processes involved in cellular senescence. The increased apoptosis of senescent cells, coupled with enhanced autophagic and mitophagic flux, allowed for the clearance of damaged organelles and the renewal of healthy cells, thereby promoting a more youthful cellular environment. Furthermore, the study demonstrated that anthocyanins improved mitochondrial function in senescent cells by increasing mitochondrial biogenesis, ATP production, and antioxidant capacity. Mitochondrial dysfunction is a hallmark of aging and is closely associated with cellular senescence. By targeting mitochondrial health, anthocyanins may contribute to the prevention and treatment of age-related diseases.

In conclusion, elderberry extracts demonstrate neuroprotective and anti-aging effects, and the key mechanisms underlying them are most likely the anti-inflammatory and antioxidant properties of their polyphenolic constituents. These findings highlight the potential of elderberry extracts as natural therapeutic agents for the prevention and treatment of age-related neurodegenerative disorders associated with chronic inflammation, oxidative stress, and cellular senescence.

## 6. Neuroregenerative Activity of Bioactive Compounds Derived from *Sambucus* Plants

Neurogenesis is a complex biological process that involves the formation of new neurons de novo in the brain. In adult mammals, neurogenesis has been shown to occur primarily in two distinct regions: the subventricular zone (SVZ) of the lateral ventricles and the subgranular zone (SGZ) of the hippocampal dentate gyrus (DG). The newly generated neurons in the SVZ migrate through the rostral migratory stream to the olfactory bulb, where they integrate into the existing neural circuitry. In the DG, the neural stem cells in the SGZ give rise to new granule cells that mature and integrate into the hippocampal network. These adult-born neurons pass through multiple developmental stages, expressing specific protein markers at each stage, before fully integrating into the existing neural circuits. The process of adult neurogenesis is regulated by various factors, including environmental enrichment, physical exercise, stress, and aging. While the exact functional implications of adult neurogenesis remain an area of active research, it is thought to play a role in learning, memory, and mood regulation [76,77].

Neuroregenerative potential refers to the ability of certain compounds, therapies, or interventions to promote the regeneration, repair, or replacement of damaged or lost neural tissue. In the context of neurodegenerative diseases, such as Alzheimer’s and Parkinson’s diseases, harnessing the neuroregenerative potential of endogenous or exogenous factors could lead to the development of novel therapeutic strategies aimed at restoring neuronal function and alleviating the symptoms associated with these disorders [78]. Plant-derived bioactive compounds, particularly polyphenols, have emerged as promising candidates for promoting neuroregeneration due to their antioxidant, anti-inflammatory, and neuroprotective properties. *Sambucus*-derived compounds have shown potential in promoting neuroregeneration via their ability to promote neural stem cell proliferation and differentiation and stimulate the secretion of neurotrophic factors [22,79].

Studies have demonstrated that extracts from *Sambucus* possess the ability to promote the proliferation and differentiation of neural stem cells (NSCs). Haratizadeh et al. [80] investigated the effect of the methanolic extract of *S. ebulus* fruit on the proliferation of NSCs derived from the hippocampus of newborn rats under oxidative stress conditions induced by H_2_O_2_. The results showed that the *S. ebulus* extract increased the proliferation rate of NSCs in a dose-dependent manner compared to the control group under oxidative stress conditions, with the highest proliferation rate observed at the concentration of 500 μg/mL. The authors suggest that the antioxidant and anti-inflammatory properties of the bioactive compounds in *S. ebulus*, such as anthocyanins, polysaccharides, steroids, and lectins, may be responsible for the observed protective effects on NSCs.

Furthermore, Liu et al. [81] demonstrated that *S. williamsii* extract can induce the differentiation of pluripotent embryonic stem cells (ESCs) into neurons. Employing a three-step differentiation strategy, the researchers observed that treatment with extract led to an upregulation of the neural-related genes *Nestin* and *Tuj1* compared to the control group, exhibiting the optimal effect on inducing neuronal differentiation. Moreover, the percentage of neurons, as indicated by *Nestin* and *Tuj1* expression, progressively increased from the P1 to P3 generations upon treatment with extract, while the expression levels of the stem cell markers *Oct4* and *Sox2* decreased, suggesting a gradual commitment of the ESCs towards the neuronal lineage.

In addition to promoting neural stem cell proliferation and differentiation, bioactive compounds derived from *Sambucus* plants have also been shown to stimulate the secretion of neurotrophic factors, which play a crucial role in the survival, growth, and differentiation of neurons in the central nervous system. Suh et al. [82] conducted a study on the chemical constituents of the twigs of *S. williamsii* var. coreana and their biological activities. The authors isolated and characterized six new iridoid glycosides, named sambucusides A-F, along with two known derivatives, and evaluated their neuroprotective effects. Interestingly, the authors assessed the neuroprotective activities of the isolated compounds by measuring their ability to induce nerve growth factor (NGF) secretion in C6 glioma cells. NGF is a critical neurotrophic factor involved in the survival, maintenance, and regeneration of neurons in the central and peripheral nervous systems. The study found that some compounds exhibited significant NGF-releasing effects, increasing NGF levels up to 147.0% compared to the control. These findings suggest that the iridoid glycosides present in *S. williamsii* var. coreana may have potential neuroregenerative properties by stimulating the secretion of NGF. The authors further discuss the structure–activity relationships of the isolated iridoid glycosides, highlighting the importance of specific structural features, such as the presence of the 2-methylbutyroyl group at C-1, the geometry of the coumaroyl moiety, and the sugar unit, in influencing their biological activities. The study provides valuable insights into the chemical diversity and potential therapeutic applications of the iridoid glycosides found in *S. williamsii* var. coreana.

In summary, bioactive compounds derived from *Sambucus* plants have demonstrated promising neuroregenerative potential through their ability to promote neural stem cell proliferation and differentiation, as well as stimulate the secretion of neurotrophic factors. Haratizadeh et al. [80], Liu et al. [81], and Suh et al. [82] highlight the potential of these compounds in promoting neuroregeneration and provide a strong rationale for further investigation into their therapeutic applications in neurodegenerative diseases. However, additional research is necessary to elucidate the specific molecular mechanisms underlying their neuroregenerative effects and to validate their efficacy in relevant preclinical and clinical models. Furthermore, exploring the synergistic effects of various bioactive compounds present in *Sambucus* plants and their potential for combination therapy may lead to the development of more effective neuroregenerative strategies.

## 7. Neuroprotective Activity of Bioactive Compounds Derived from *Sambucus* Plants

Medicinal plants have been used for centuries in traditional medicine systems to treat various neurological disorders. The neuroprotective potential of these plants has been attributed to the presence of a wide array of bioactive compounds, including polyphenols, terpenoids, and other phytochemicals. These compounds exert their neuroprotective effects through multiple mechanisms, such as reducing oxidative stress, inflammation, and apoptosis, as well as by modulating mitochondrial function [22]. Among the various medicinal plants studied for their neuroprotective properties, those belonging to the genus *Sambucus* have garnered significant attention in recent years.

Several in vivo studies have explored the neuroprotective effects of *Sambucus* extracts and their bioactive compounds using animal models of neurodegenerative diseases. The findings provide valuable insights into their potential therapeutic applications with regard to cognitive function, motor performance, neuronal survival, oxidative stress, inflammation, and apoptosis (Table 1).

Additional in vivo studies have further explored the neuroprotective potential of *Sambucus* extracts in humans with mild cognitive impairment (MCI). Curtis et al. conducted a randomized, double-blind, placebo-controlled trial examining the effects of elderberry juice on cognition and inflammatory markers in patients with MCI. Participants consumed either elderberry juice or a placebo three times daily for 6 months. The study found a trending improvement in visuospatial cognitive flexibility for the elderberry group compared to the placebo group at 6 months. Furthermore, the elderberry condition showed significant or trending decreases over time in several markers of low-grade peripheral inflammation, including vasorin, prenylcysteine oxidase 1, and complement Factor D. In contrast, the placebo condition showed increases in some inflammatory markers over time. These findings suggest that elderberry juice consumption may provide some cognitive benefits and reduce low-grade inflammation in individuals with MCI, supporting the potential neuroprotective effects observed in preclinical models [87].

The in vivo studies discussed above have provided valuable insights into the potential mechanisms underlying the neuroprotective effects of *Sambucus* extracts and their bioactive compounds in neurodegenerative diseases. These mechanisms involve modulation of inflammatory pathways, apoptotic signaling, mitochondrial function, and oxidative stress.

One of the key routes by which *Sambucus* extracts exert their neuroprotective properties is by attenuating neuroinflammation. *Sambucus* compounds have been shown to reduce microglial activation and the expression of pro-inflammatory cytokines such as TNF-α and IL-1β in animal models of PD and AD. This anti-inflammatory effect may be mediated through the inhibition of NF-κB and MAPK signaling pathways, which are known to regulate the production of pro-inflammatory mediators [88,89].

They are also believed to act by modulating apoptotic signaling. *Sambucus* extracts have been found to reduce the levels of apoptotic markers such as caspase-3 in animal models of neurodegenerative diseases. This anti-apoptotic effect may be achieved by promoting neuronal survival through the regulation of pro-apoptotic and anti-apoptotic proteins. Interestingly, *Sambucus*-derived compounds have also been found to modulate apoptotic signaling in brain tumor cells. Lamy et al. [90] investigated the antiproliferative activity of elderberry and elderflower extracts from two Canadian cultivars against human glioma and brain microvascular endothelial cells under normoxic and hypoxic conditions. The extracts inhibited cell proliferation in a dose-dependent manner, with the berry extracts being more potent than flower extracts. The antiproliferative effects were mediated by cell cycle arrest and apoptosis induction, with specific anthocyanins and other polyphenols likely working in synergy. These findings suggest that while *Sambucus* extracts may promote neuronal survival in neurodegenerative diseases by reducing apoptosis, they may also exert antitumor effects in brain cancer cells by inducing apoptosis. This dual role of *Sambucus*-derived compounds in modulating apoptotic signaling highlights their potential as neuroprotective and chemopreventive agents.

Moreover, mitochondrial dysfunction is a common feature of neurodegenerative disorders, and *Sambucus* compounds have demonstrated the ability to improve mitochondrial function. For instance, SC-Nanophytosomes containing elderberry anthocyanins enhanced respiratory control rate, increased the activity of individual respiratory complexes, and improved the fatty acid profile of membrane phospholipids in a rat model of PD. These findings suggest that *Sambucus* compounds may exert their neuroprotective effects by maintaining mitochondrial integrity and function.

In addition to their beneficial effects on mitochondrial function in neurodegenerative disorders, *Sambucus* compounds have also been shown to mitigate mitochondrial dysfunction in other pathological conditions. Fathi et al. [91] investigated the antiemetic and neuroprotective properties of *S. ebulus* fruit extract in the context of oxidative damage induced by retching in young chickens. The study revealed that retching induced by ipecac and copper sulfate resulted in oxidative stress and protein oxidation in the brain mitochondria, as evidenced by increased lipid peroxidation and protein carbonyl levels. Treatment with the methanolic extract of *S. ebulus* fruit effectively reduced lipid peroxidation levels, demonstrating its protective effect against oxidative damage. Furthermore, the extract was found to modulate catalase activity and protein carbonyl content, suggesting its ability to regulate antioxidant defenses and prevent protein modifications induced by retching. Notably, at a dose of 100 mg/kg, the *S. ebulus* extract significantly improved mitochondrial function, highlighting its neuroprotective potential. The authors attribute these beneficial effects to the phenolic and flavonoid content of the extract, which possess antioxidant properties.

In addition, oxidative stress is another major contributor to neurodegeneration, and *Sambucus* extracts have shown potent antioxidant properties. In animal models of Alzheimer’s disease and Parkinson’s disease, *Sambucus* compounds reduced reactive oxygen species levels, enhanced glutathione content, and restored the activity of antioxidant enzymes. The activation of the Nrf2-mediated antioxidant response pathway has been identified as a potential mechanism for the antioxidant effects of *Sambucus* compounds [92].

In conclusion, the neuroprotective effects of *Sambucus* compounds can be attributed to their ability to modulate multiple signaling pathways involved in inflammation, apoptosis, mitochondrial function, and oxidative stress. These mechanisms work in concert to promote neuronal survival, reduce neuroinflammation, and maintain cellular homeostasis in the face of neurodegenerative insults. The in vivo studies provide compelling evidence for the neuroprotective effects of *Sambucus* extracts and their bioactive compounds in animal models of neurodegenerative diseases such as AD and PD. The observed benefits include improvements in cognitive function, motor performance, neuronal survival, and mitigation of oxidative stress, inflammation, and apoptosis. These findings suggest that *Sambucus*-derived compounds may have potential therapeutic applications in the prevention and treatment of neurodegenerative disorders. However, further research is needed to elucidate the specific molecular targets and signaling cascades involved in their neuroprotective actions, which may lead to the development of novel therapeutic strategies for neurodegenerative diseases.

## 8. *Sambucus* as a Functional Ingredient for Food

*Sambucus* has emerged as a valuable functional ingredient in the food industry due to its unique nutritional profile, sensory properties, and content of bioactive compounds, particularly anthocyanins. The fruits and flowers are widely used in a diverse range of food products, including juices, syrups, jams, jellies, pies, desserts, and alcoholic beverages. The versatility of *Sambucus* as a food ingredient can be attributed to its rich nutrient composition and distinctive sensory characteristics [93,94]. Moreover, elderberry pomace, a byproduct of juice production, is a valuable source of anthocyanins and is utilized for the production of extracts, lyophilized dyes, animal feed, and organic fertilizers [95]. However, further research is necessary to fully understand its potential applications, the stability of its bioactive compounds during processing, and the impact of various factors such as cultivar, ripeness, and environmental conditions on its chemical composition [96,97,98]. Furthermore, the presence of toxic cyanogenic glycosides in elderberry necessitates the development of effective strategies to reduce or eliminate these harmful substances while retaining the beneficial compounds [99].

The vibrant colors of elderberry fruits and flowers have garnered significant attention as potential sources of natural food colorants. The increasing demand for clean-label and natural ingredients in the food industry has driven research into the coloring properties and stability of elderberry extracts. Elderberry fruits are particularly rich in anthocyanins, the water-soluble pigments responsible for their deep purple hue. The primary anthocyanins identified in elderberries are cyanidin-3-*O*-sambubioside and cyanidin-3-*O*-glucoside, which together account for approximately 85–90% of the total anthocyanin content [100,101]. Other anthocyanins present in smaller quantities include cyanidin-3-O-sambubioside-5-*O*-glucoside, cyanidin-3,5-*O*-diglucoside, and cyanidin-3-*O*-rutinoside [93,101]. The anthocyanin profile of elderberries has been shown to vary among different cultivars and genotypes [102]. Environmental factors such as growing conditions, ripeness, and climatic conditions can also influence the anthocyanin composition [103]. Despite these variations, elderberries consistently exhibit higher anthocyanin content compared to other sources [104].

Elderberry extracts have been successfully incorporated as natural colorants in various food products, including yogurt [105], kefir [106], meat products [107], and baked goods [96,103]. The addition of elderberry juice or extract not only imparts an appealing red-purple color but also enhances the nutritional value and antioxidant properties of the food matrix. For instance, croissants colored with elderberry juice displayed high antioxidant capacity and bioactive compound content [101]. Similarly, the incorporation of elderberry powder in gluten-free wafers increased their polyphenol and mineral content [108]. The inclusion of elderberry extracts in food products not only improves their visual appeal but also provides additional health benefits to consumers.

The stability of elderberry anthocyanins is a critical factor in their successful application as food colorants. Anthocyanins are sensitive to various environmental factors such as pH, temperature, light, and the presence of oxygen, which can lead to their degradation and loss of color [109]. To address these stability challenges, researchers have explored different strategies to improve the stability and bioavailability of elderberry anthocyanins. One promising approach is the use of encapsulation techniques, which involve entrapping the anthocyanins within a protective matrix or carrier material. Encapsulation methods such as spray-drying, freeze-drying, and liposomal encapsulation have been shown to enhance the stability and bioavailability of anthocyanins [110]. For instance, Akhavan Mahdavi et al. demonstrated that the encapsulation of elderberry anthocyanins in chitosan nanoparticles significantly improved their stability under various storage conditions and increased their bioaccessibility in an in vitro digestion model [111]. The development of effective encapsulation strategies can help overcome the stability limitations of elderberry anthocyanins and expand their potential applications as natural food colorants.

One of the most common applications of elderberry is in the production of functional beverages. Vujanović et al. [112] developed a novel elderberry juice rich in anthocyanins, phenolic acids, and flavonoids, which exhibited strong antioxidant and enzyme inhibitory activities. The authors suggested that this juice could be used as a functional ingredient to prevent oxidative stress and metabolic disorders. Similarly, Schmitzer et al. [113] investigated the production of elderberry wine, which was found to contain high levels of anthocyanins, quercetin derivatives, and phenolic acids; the wine showed antioxidant potential comparable to red wines, indicating its potential as a functional alcoholic beverage with health benefits. In addition to their use in functional beverages, *Sambucus* extracts have also been incorporated into dietary supplements. The high content of bioactive compounds in elderberry, particularly anthocyanins and other polyphenols, has led to its use in the formulation of various health-promoting supplements [114].

The processing of elderberry can significantly impact the content of valuable compounds in the final product. Various processing techniques, such as drying, heating, and filtration, can affect the stability and concentration of bioactive compounds, particularly anthocyanins and polyphenols. Szalóki-Dorkó et al. [115] investigated the effects of different processing steps involved in elderberry concentrate production on the anthocyanin and polyphenol content of several elderberry varieties. The study revealed that the processing steps, especially heat treatment and microfiltration, had significant effects on the levels of these bioactive compounds. Interestingly, the extent of the impact varied among the different elderberry varieties studied. Some varieties showed greater retention of anthocyanins and polyphenols during processing compared to others. The authors emphasize the importance of considering varietal differences when selecting elderberries for industrial processing. They suggest that the choice of elderberry variety should be based on the desired bioactive compound profile and the stability of these compounds during processing. By selecting varieties with high levels of heat-stable anthocyanins and polyphenols, manufacturers can optimize the retention of health-promoting compounds in the final elderberry products. This approach can help ensure the production of high-quality elderberry-based functional ingredients with consistent bioactive properties.

The potential of elderberry extracts in developing edible films and coatings for food applications has also been explored. Ribeiro et al. [116] developed edible films incorporating polyphenols extracted from elderberry using different biopolymers, namely modified chitosan, sodium alginate, and gum arabic. The elderberry-enriched films demonstrated high entrapment efficiency for polyphenols, ranging from 74.0% to 99.9%, and exhibited controlled release properties that were dependent on the specific biopolymer matrix. These findings suggest that elderberry-enriched edible films could serve as a promising alternative to synthetic packaging materials, offering both protective and health-promoting functionalities.

When considering the application of *Sambucus* extracts in food and therapeutic products, it is crucial to evaluate their safety and potential toxicity. Several studies have investigated the cytotoxic and genotoxic effects of elderberry and elderflower extracts. Bratu et al. [117] assessed the cytotoxic and genotoxic properties of *S. nigra* fruit extract powder using the Allium test. The aqueous solutions of the extract exhibited minor cytotoxic effects, characterized by mitodepressive activity and inhibition of mitosis at the preprophase stage. The mutagenic effect was found to be concentration-dependent and influenced by the duration of treatment. The study concluded that elderberry fruit extract powder solutions exhibited mutagenic activity at higher concentrations (1 g/dL), while no mutagenic effects were observed at lower concentrations (0.1 g/dL). In another study, Banach et al. [118] evaluated the cytotoxic properties of two batches of elderberry dry extracts on various cancer cell lines (A-549, A-2780, MCF-7, Caco-2) and peripheral blood mononuclear cells (PBMCs). The extracts demonstrated differential cytotoxic potentials towards the tested cell lines, with the lowest IC50 values observed for the cancerous cell lines (HeLa and HT29) and the highest for the normal cell line (L929), indicating low toxicity. Interestingly, both extracts stimulated the proliferation of PBMCs, suggesting potential immunostimulatory effects.

Furthermore, Ferreira-Santos et al. [119] investigated the impact of gastrointestinal digestion on the bioactivity and toxicity of *S. nigra* flower and berry extracts. The digested and non-digested extracts exhibited significantly different effects on various cell lines. The IC_50_ values were highest for the normal cell line (L929), indicating low toxicity, while lower values were observed for the cancerous cell lines (HeLa and HT29). An in vivo Artemia salina lethality bioassay revealed a dose-dependent effect of the extracts, with the digested berry extract inducing the lowest mortality rate. These findings highlight the significant influence of the gastrointestinal digestion process on the bioactivity and toxicity profiles of elderberry and elderflower extracts, emphasizing the need for further in vivo studies to fully understand their safety and efficacy.

In conclusion, *Sambucus* has emerged as a promising functional ingredient in the food industry due to its unique nutritional profile, sensory properties, and rich content of bioactive compounds, especially anthocyanins. Elderberry extracts have been successfully incorporated as natural food colorants, functional ingredients in beverages and dietary supplements, and in the development of edible films and coatings. However, the stability of elderberry anthocyanins during processing remains a challenge, and further research is needed to optimize extraction and encapsulation techniques. Additionally, while studies have investigated the cytotoxicity and genotoxicity of elderberry extracts, more in vivo studies are required to fully understand their safety and efficacy. As interest in natural and functional food ingredients continues to grow, elderberry presents a valuable opportunity for the food industry to develop innovative and health-promoting products. Future research should focus on exploring novel applications, improving the stability of bioactive compounds, and conducting comprehensive safety assessments to ensure the successful incorporation of elderberry into a wider range of food products.

## 9. Conclusions

This review highlights the significant neuroprotective potential of bioactive compounds derived from the *Sambucus* genus, mainly *S. nigra*, and their promising applications as functional food ingredients for promoting brain health and cognitive performance. The antioxidant, anti-inflammatory, anti-aging, and neuroregenerative properties of *Sambucus*-derived compounds have been demonstrated in various models of neurodegenerative diseases. A registered clinical trial (NCT02414607) aims to investigate the effects of elderberry juice on cognitive decline in subjects with mild cognitive impairment, building upon existing evidence supporting the anti-inflammatory and antioxidant effects of elderberry preparations. The incorporation of *Sambucus*-derived bioactive compounds into functional foods and dietary supplements presents a promising approach to harness their neuroprotective potential. However, further research is necessary to address the challenges associated with their stability, bioavailability, and sensory properties in food matrices, as well as to validate their efficacy and safety in human populations. The evidence presented in this review supports the use of *Sambucus*-derived bioactive compounds as a promising dietary strategy to promote brain health and prevent age-related cognitive decline.

## Figures and Tables

**Table 1:** Summary of in vivo studies investigating the neuroprotective effects of *Sambucus*-derived compounds in models of neurodegenerative diseases.

Species	Animal Model	*Sambucus* Treatment	Dose of the *Sambucus* Extract	Duration of Treatment	Neuroprotective Effects	Mechanisms	Ref.
*Sambucus nigra*	Rat model of Alzheimer’s disease	Elderberry-enriched diet	2%	8 weeks	Improved memory and learning functions Alleviated astrogliosis and astrocyte reactivity Reduced apoptosis and neuronal degeneration Preserved spatial distribution of hippocampal neurons	Antioxidant Anti-inflammatory (decreased TNF-α and IL-1β) Anti-apoptotic (decreased caspase-3)	[83]
*Sambucus nigra*	Rat model of Alzheimer’s disease	Elderberry-enriched diet	2%	8 weeks	Improved spatial memory, learning, and long-term memory Prevented neuronal degeneration in the hippocampus Increased neuronal density and decreased number of degenerated neurons	Not specified	[84]
*Sambucus nigra*	Rat model of Parkinson’s disease	Elderberry-enriched diet: SC-Nanophytosomes (elderberry anthocyanins and marine algae polar membrane lipids)	2.5 mg/mL	3 weeks	Improved motor symptoms Normalized α-synuclein levels Restored antioxidant enzyme activity in brain regions	Attenuated mitochondrial dysfunction Enhanced respiratory control rate Increased activity of individual respiratory complexes Improved fatty acid profile of membrane phospholipids	[85]
*Sambucus nigra*	Rat model of Huntington’s disease	Elderberry diet	2%	8 weeks	Improved motor coordination, locomotion and muscle activity Prevented striatal volume reduction and neuronal loss Attenuated microglial activation and neuroinflammation Reduced apoptotic marker caspase-3 and pro-inflammatory cytokine TNF-α Decreased reactive oxygen species and enhanced glutathione content in the striatum	Anti-inflammatory Anti-apoptotic Antioxidant	[86]

## Data Availability

Not applicable.
